# Building EVA (Educación, Vinculación, y Autoayuda): Tutorial for the Development of a Digital Mental Health Chatbot for Adolescents Living With HIV

**DOI:** 10.2196/75351

**Published:** 2026-04-28

**Authors:** Diego Humberto Vasquez, Neil Rupani, Carmen Contreras, Lenka Kolevic, Molly Forrest Franke, Jerome Timothy Galea

**Affiliations:** 1 Socios En Salud Sucursal Peru, Lima, Peru; 2 Morsani College of Medicine, University of South Florida, Tampa, FL, United States; 3 School of Social Work, College of Behavorial and Community Sciences, University of South Florida, 13301 Bruce B Downs Blvd, MHC 1400, Tampa, FL, 33612-3807, United States, 1 813 974 2310; 4 Instituto Nacional de Salud del Niño, Lima, Peru; 5 Departamento Académico de Pediatría, Universidad Nacional Mayor de San Marcos, Lima, Peru; 6 Department of Global Health and Social Medicine, Harvard Medical School, Harvard University, Boston, MA, United States

**Keywords:** chatbot, mHealth, adolescent, HIV, mental health, depression, mobile health

## Abstract

Adolescents living with HIV face higher rates of mental health morbidity compared to other age groups, particularly depressive symptoms, while access to specialized services remains limited in many low- and middle-income countries. Chatbots offer a promising, low-cost approach to delivering structured mental health support in resource-constrained settings; however, practical guidance on how to develop such tools with end-user involvement remains scarce. This tutorial provides a step-by-step guide to the human-centered design and development of a mental health chatbot for adolescents living with HIV, illustrated through the creation of EVA (*Educación, Vinculación, y Autoayuda*), a chatbot cocreated with adolescents living with HIV in Peru. Guided by human-centered design principles, the tutorial covers three key steps: (1) understanding user needs through qualitative research, (2) iterative chatbot development with a Youth Advisory Board across five structured sessions, and (3) external review and testing before launch. Throughout the process, adolescents contributed to defining the chatbot’s tone, visual identity, navigation structure, and content priorities. The chatbot was built using a low-cost messaging platform and incorporated multimedia components, including brief animated videos, to enhance engagement. Throughout development, iterative testing with the Youth Advisory Board and external health care professionals informed refinements in usability, content clarity, emotional tone, and accessibility. This tutorial synthesizes key methodological decisions, lessons learned, and challenges encountered, providing practical guidance for researchers and practitioners seeking to develop similar adolescent-centered digital mental health tools in low- and middle-income countries. Key takeaways include the importance of early and sustained adolescent involvement, the value of iterative prototyping, and the feasibility of building functional chatbots with limited resources to help address the health service gap experienced by adolescents living with HIV.

## Introduction

In 2024, approximately 1.57 million adolescents aged 0-19 years were living with HIV worldwide, accounting for about 11% of new HIV infections [1]. Adolescents living with HIV face unique challenges that place them at greater risk of mental health problems compared to their peers without HIV, as well as to children and adults living with and without HIV [2,3]. The coexistence of a chronic, potentially life-altering illness with the pressures of transitioning from pediatric to adult care settings can heighten psychological distress, undermine adherence to antiretroviral therapy (ART), and compromise viral suppression and overall health [4-6]. These vulnerabilities are reflected in the high prevalence of depression among adolescents living with HIV, estimated at nearly 26%, which is markedly higher than the rate observed in the general adolescent population, where fewer than 10.5% experience depression [7,8].

Left unaddressed, mental health problems—especially depression and anxiety—among adolescents living with HIV are associated with suboptimal adherence to ART, leading to negative treatment outcomes [9]. At the same time, however, there is a global shortage of mental health services, with an estimated 70% of people in need worldwide lacking access [10]. For adolescents living with HIV, who predominantly live in low- and middle-income countries (LMICs), this gap results in a severe lack of mental health services, which can, in turn, impact HIV treatment outcomes and overall health.

Multiple factors at the personal, health care system, and policy levels undermine adolescents living with HIV’s access to mental health services. Stigma around HIV and mental health can discourage adolescents from disclosing symptoms or seeking care [11,12], while dependence on caregivers adds logistical and privacy concerns [13]. Mental health services are typically not integrated with HIV care, and facilities may lack trained personnel, resulting in delayed or suboptimal care [14]. In many LMICs, including Peru, a shortage of mental health care professionals, limited financial resources, and insufficiently integrated policies further constrain access to adolescent-focused interventions [15,16].

Against this backdrop, depression emerges as one of the most common and pressing mental health concerns among adolescents living with HIV [17]. Although many such adolescents experience mild to moderate symptoms rather than severe depression, even low-intensity symptoms can escalate into more serious issues if left unaddressed [6,18]. Indeed, preliminary data in Peru indicate that up to 92% of adolescents living with HIV presenting with depression have symptoms in the mild-to-moderate range [19]. These youth receive few—if any—routine mental health interventions, such as education, self-help strategies, or linkage to community-based resources, missing a critical opportunity to prevent progression to severe depressive episodes and further ART nonadherence [20].

Given these converging challenges, digital solutions have gained traction to fill the “presevere depression service gap” [21]. Chatbots—automated conversational agents—show promise for delivering accessible, anonymous, and scalable mental health support, requiring no special software and operating via widely used platforms such as SMS text messaging, WhatsApp (WhatsApp Inc), and Facebook Messenger (Meta Platforms, Inc) [21,22]. In Peru, chatbots facilitated mass depression screening, reaching more than 20,000 adults during the COVID-19 pandemic [23,24], suggesting the potential for a chatbot tailored to the needs of adolescents living with HIV that offers psychoeducation, coping strategies, and linkage to care.

Developing such a chatbot, however, requires active end-user participation to maximize relevance and acceptability. Human-centered design (HCD)—also known as participatory or user-centered design—integrates end-user voices at every stage, including content creation, prototype testing, and refinement [25]. Previous digital mental health interventions have experienced high attrition rates among young users when they fail to address factors such as cultural context, stigma, and interface usability [26]. By involving adolescents living with HIV from the outset, we can better tailor the chatbot’s functions and content, improving engagement and mitigating the risk of dropout [27]. Yet, despite the growing popularity of chatbots in health care, few published studies describe a step-by-step approach to applying HCD in chatbot development.

**Figure 1.**
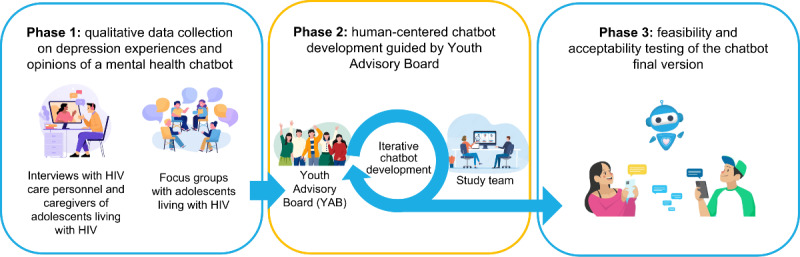
Phases of the principal study.

This tutorial describes the development of EVA (*Educación, Vinculación, y Autoayuda;* in English: Education, Linkage to Care, and Self-Help), a chatbot created during August-December 2023 as part of a broader mixed-methods study assessing the feasibility of chatbots for Peruvian adolescents living with HIV [20], to illustrate a practical, replicable approach to mental health chatbot development. The larger study comprised three phases (Figure 1): (1) qualitative data collection to understand how adolescents living with HIV conceptualized depression and their initial views and opinions of a mental health chatbot, (2) human-centered development of the chatbot (focus of this tutorial), and (3) feasibility and acceptability testing of the chatbot.

## Ethical Considerations

The principal study, which includes the procedures described here, was reviewed and approved by an Institutional Bioethics Committee (Comité Institucional de Bioética) of VÍA LIBRE in Peru. The Institutional Review Board (IRB) approval number is CIB No 8591 (2023a).

The IRB at the University of South Florida formally agreed to rely on VÍA LIBRE for the review, approval, and continuing oversight of the research project under an interagency IRB Authorization Agreement (University of South Florida IRB study no 005124). For participants aged younger than 18 years, informed assent was obtained as well as informed consent from their legal guardians. For participants older than 18 years, informed consent was obtained.

## Tutorial Objectives

In this tutorial, we outline our human-centered approach to creating a novel chatbot designed to deliver low-intensity, supportive interventions for adolescents living with HIV in Peru experiencing mild to moderate depressive symptoms, as defined by commonly used clinical instruments such as the Patient Health Questionnaire-9 (PHQ-9). Specifically, this tutorial aims to (1) describe how HCD can be meaningfully applied with adolescents throughout the development of a digital mental health tool; (2) explain how chatbot modules were structured to deliver mental health information and supportive content in a fluid, engaging, and well-organized manner; (3) illustrate how iterative review cycles with a Youth Advisory Board (YAB) can be conducted to refine content, tone, usability, and cultural relevance; and (4) describe how ethical considerations, multimedia elements, and clinical validation processes can be integrated into chatbot development.

## Chatbot Development: Key Considerations

### HCD Methodology

Chatbot development should follow HCD methodology. HCD focuses on designing conversational experiences centered on users’ needs, expectations, and emotions. Its iterative approach enables the creation of more intuitive, efficient, and empathetic chatbots by adhering to 4 main principles: being people-centered, solving the right problem, considering everything as a system, and making simple and small interventions [28].

Central to this approach is co-design, which positions end users not merely as participants but as active contributors to decision-making, ensuring that the intervention reflects their realities, language, and priorities. To operationalize this participatory framework, a YAB composed of 6 Peruvian adolescents living with HIV was established to guide chatbot development.

### Ethical Safeguards of the Design

Designing a mental health chatbot for adolescents—particularly adolescents living with HIV—requires integrating ethical, privacy, and clinical safeguards from the earliest stages of development. These considerations are not secondary features but core design components that directly influence trust, safety, and acceptability.

- Privacy and anonymity: the EVA chatbot was designed to minimize personal data collection, following recommendations from adolescents living with HIV consulted before the design phase, which were later reinforced and validated by the YAB. Users were only asked to enter a first name or nickname, and no information regarding HIV status, treatment history, or other personal identifiers was requested at any point. This approach reduces barriers to engagement and is especially important in contexts where stigma related to HIV or mental health remains high. User data were stored securely on Smartbot360 servers under the chosen display name, in a system that is the Health Insurance Portability and Accountability Act–compliant and suitable for sensitive health data [29].
- Transparency about the chatbot’s nature: upon initiation, the chatbot clarified that it was a virtual tool and not a human being. Explicitly communicating the system’s nonhuman nature is an important ethical practice, helping prevent misunderstanding about the type of support provided.
- Boundaries of support and linkage to care: because chatbots are not substitutes for professional care, EVA incorporated a linkage module. After addressing topics such as anxiety, depression, and eating-related concerns, users were asked whether they wished to connect with a health care professional. Communication through the linkage module occurred entirely in written form within the chatbot interface, without requiring personal identifiers. All conversations between users and health care professionals were securely recorded on Smartbot360 servers and were accessible only to the study team.
- Clinical accuracy and destigmatizing language: the chatbot’s educational content was adapted from reliable international sources, including the World Health Organization (WHO) and the Joint United Nations Program on HIV/AIDS (UNAIDS), and reviewed by the YAB, research team, as well as external health care professionals with experience in adolescent HIV care, ensuring accuracy and the use of appropriate, destigmatizing language.
- Informed consent considerations: when developing similar tools, teams should decide early whether consent will be handled digitally, in person, or through a hybrid model. For EVA, digital informed consent was not incorporated into the chatbot structure during development, as a printed informed consent document was planned for phase 3 of the principal study. During that testing phase, participants will also receive detailed instructions and guidance from a mental health care professional, who will supervise the chatbot’s use and ensure that all ethical considerations are addressed.

### Study Team and Software Tools

The development team should include a mix of health care professionals, researchers, and technical staff with complementary expertise. For EVA, the team included 4 health care professionals: 2 psychologists (CC and DHV), a mental health researcher and clinician with experience in chatbot development (JTG), and a medical student (NR). DHV and NR were primarily responsible for software development, while CC and JTG oversaw the content and construction of the chatbot.

The chatbot was developed using Smartbot360 software [30]. JTG used this software for chatbot development in previous studies aimed at developing tools to improve mental health for other populations [31,32]. In June 2023, JTG conducted virtual and face-to-face training sessions on the use of Smartbot360.

## Building EVA, Step-by-Step

### Step 1: Understanding User Needs

The first step should involve collecting information on the perceptions, acceptability, and feasibility of a mental health chatbot among the target adolescent population and the adults involved in adolescents’ care, to gather preliminary insights. For EVA, we conducted 5 focus groups with 28 adolescents living with HIV, complemented by interviews with health care professionals providing pediatric HIV services, and caregivers of adolescents living with HIV (for details of this step, refer to [33]). Some examples of questions from this phase include “What opinions do you have about the idea of a mental health chatbot?” and “What would be the barriers or difficulties to accessing a chatbot (access to a device, internet, etc)?” These questions were derived from Sekhon’s theoretical framework of health care intervention acceptability [34].

The preliminary findings on chatbot acceptability provided valuable insights into users’ expectations and perceived challenges before development. Participants emphasized the importance of a secure, confidential, and easily accessible tool, which they believed could serve as an educational resource to strengthen self-help skills, given the long wait times for professional care in public health services, which can range from weeks to months. Therefore, the chatbot’s mobile-friendly design was seen as advantageous, given that adolescents naturally engage with technology [35].

At the same time, potential drawbacks or challenges of a chatbot can be identified early. Some adolescents felt that a chatbot could be perceived as impersonal, artificial, and with limited emotional scope for dialogue on sensitive topics that teens may wish to discuss. Furthermore, adolescents’ limited access to the internet and mobile devices needs to be considered, as many have limited resources. There were mixed views on the degree of parental involvement, with teens concerned about who might have access to the information they enter and whether parents or guardians would be monitoring their conversations. Adolescents’ familiarity with technology could subsequently become a challenge. If they find the chatbot uninteresting or unentertaining, they are unlikely to use it, despite the useful information it may contain.

With these strengths and challenges in mind, the chatbot’s structure and key functions can be defined. Insights from the focus groups can guide decisions on the most appropriate access platform (eg, web-based vs social media). For us, it became evident that the most viable option for accessing the chatbot would be a web-based interface rather than social media or WhatsApp, given ethical, privacy, and development resource considerations.

### Step 2: Human-Centered, Iterative Development Sessions

#### Overview

To identify key characteristics that can improve the acceptability of a chatbot for adolescents, it is essential to collaborate directly with this population. For this reason, we convened a YAB comprised of adolescents representative of the end-user population. YAB members can be selected from Step 1 participants (in our case, adolescents living with HIV), prioritizing individuals who were particularly engaged during focus groups—those who openly shared their opinions, demonstrated an understanding of adolescent mental health, and recognized the challenges faced by other adolescents living with HIV while adhering to treatment. Small advisory groups are recommended to be diverse yet compact enough to ensure meaningful participation and sustainability [36,37], and research suggests that groups of 5-7 members optimize discussion, problem-solving, and consensus building [38]. Ultimately, our YAB consisted of 4 females and 2 males, aged 11-19 years (Table 1).

**Table 1.** Sociodemographic characteristics of adolescents on the Youth Advisory Board (YAB).

Characteristic	Age (11-14 years)	Age (15-19 years)	Total
Sex			
Male	2	2	4
Female	0	2	2
Sexual orientation			
Heterosexual	2	2	4
Homosexual	0	2	2
HIV acquisition			
At birth	0	2	2
Sexually	2	2	4
Parents who died from HIV			
At least one	1	1	2
None	1	3	4

The YAB’s primary task should be to test the chatbot during in-person sessions, providing feedback on its design, content, and functionality, and suggesting ideas for improvement. Sessions should be held at regular intervals, allowing sufficient time for the team to discuss, develop, and test changes between sessions. Each session should last 90‐120 minutes, including breaks, in accessible, comfortable locations for participants.

Adolescents should access the chatbot individually on their own smartphones, when possible, while additional devices can be provided for those without access. After the interaction period, participants should share their recorded opinions and discuss points of agreement and disagreement to identify key modifications for the next version of the chatbot. With guidance from a facilitator, the YAB can then proceed to evaluate the next feature or content area selected for that session. Structured feedback tools may also be incorporated. For example, we developed a Likert-scale questionnaire (1=very dissatisfied and 5=very satisfied) to assess satisfaction with videos, usefulness and clarity of information, ease of use, and overall experience, with additional items added to evaluate specific modules such as self-help activities or pathways for seeking professional support.

Plan a series of group sessions spaced approximately 30 days apart, scheduled at times convenient for adolescent participants. Sessions should be held in a safe, youth-friendly environment. For EVA, we conducted 5 group sessions between August 2023 and January 2024. All sessions took place on Saturdays to accommodate participants’ availability and were held at a nongovernmental organization that promotes health among the lesbian, gay, bisexual, transgender, queer or questioning, intersex, and asexual populations through research and programs focused on the prevention, diagnosis, and treatment of sexually transmitted infections, HIV, and AIDS. During the sessions, YAB members accessed the chatbot through a web link and spent an average of 20 minutes interacting with the content selected for that session, writing down their impressions and ideas on paper.

Because YAB meetings can generate a wide range of ideas and expectations, teams should prioritize refining prototypes to enhance functionality and implement practical solutions to address challenges and suggestions that emerge during the development process.

#### Session 1: Foundations of Chatbot Development

The first YAB session should introduce the study and foster participant engagement. Group activities, such as icebreaker exercises to introduce the YAB and study team members, and informal conversations, can help to build rapport and establish a trusting environment that encourages adolescents to share opinions, offer critiques, and stay meaningfully involved throughout chatbot development. Establishing trust and supportive group dynamics has been shown to enhance engagement and make digital health interventions more relatable and trustworthy to young people when they are actively involved in cocreation processes, improving the relevance and impact of the resulting resource [39].

The session should also provide an overview of the study goals and planned activities for future meetings and introduce preliminary concepts related to the chatbot’s content, its proposed functionality, and structural design, to gather adolescents’ perspectives and feedback before developing the first version. The session may also include opportunities for the YAB to shape visual and identity-related elements, such as the chatbot’s name and avatar. Inviting adolescents to name the chatbot is especially valuable, as it can strengthen their sense of ownership in the development process.

A brainstorming activity should be conducted with the YAB to discuss key chatbot features, such as confidentiality, health content, and ideas for making the chatbot engaging for adolescent users. For EVA, the YAB provided feedback on the chatbot’s avatar design (a graphic character representing the chatbot), reviewed the script for an introductory video, and decided to name the chatbot “EVA,” in accordance with its main functions.

An important early activity in chatbot development is creating a flowchart that maps the chatbot’s structure to its core themes and objectives. Once key modules are defined, content can be added based on each module’s purpose. The first chatbot prototype, developed after Session 1, should prioritize structure over visual polish or complex interactions, allowing the YAB to explore how the chatbot communicates and what types of interactions are possible. Even a simple version helps adolescents better understand the tool’s potential than explanations or external examples alone. Including basic but functional content enables users to navigate the system and provide informed feedback on information needs, usability, interaction styles, and content organization, which can then be refined in later iterations.

After the first session, our team developed an initial web-based prototype EVA (version 0.1) guided by YAB priorities and with a strong focus on confidentiality: the chatbot only asked users to enter a name and did not store conversations on their devices. Upon entry, 3 buttons directed users to the EVA modules. The “Education” module allowed users to select topics or type queries, though content was limited to 8 mental health and HIV-related terms. The “Linkage to Care” module simulated real-time written communication with a study team member as an example of connection to professional support, while the “Self-Help” module was not yet active.

#### Session 2: Testing the EVA Prototypes

After a workable, limited-feature prototype chatbot is developed, repeat consultation with the advisory board is essential. Two important reasons for early consultation with the advisory board, albeit with an underdeveloped chatbot, are to confirm the core features of the chatbot planned during Step 1 and to strengthen the collaborative relationship with the advisory board by demonstrating how their input shaped the chatbot programming (and explaining why some features could not be included). For the first chatbot testing experience with a YAB, ensure that access to the chatbot is simple and reliable. Provide direct entry (eg, via web link) and verify beforehand that the platform runs smoothly on common mobile devices. Facilitators should ideally be members of the development team so they can monitor real-time use, identify navigation or performance issues, and resolve technical problems immediately. Stable Wi-Fi access is essential to prevent connectivity disruptions, and backup devices (eg, smartphones or tablets) should be available for participants whose personal devices are incompatible or have limited functionality.

Although adolescents should be given time for independent exploration proportional to the amount of content under review, the first session should also include a guided walkthrough. Early prototypes are often not fully intuitive, so facilitators should briefly explain the purpose, structure, and function of each module before or during use. This helps align expectations and ensures that feedback reflects the tool’s intended design rather than confusion about navigation. The criteria for which suggestions to implement, adapt, or exclude should consider the development team’s technical capacity, available time, and financial resources, and the relevance of each modification to the intervention’s goals.

For EVA’s second session, YAB members accessed EVA (version 0.1) via a web link and explored it for 20 minutes. Afterward, technical issues were documented, and group feedback was collected on content and functionality. Adolescents confirmed that only essential information, such as name and age, should be required to access the chatbot, avoiding sensitive data such as phone numbers and ensuring that conversations are deleted after use.

For content, the YAB prioritized anxiety and depression as key topics and suggested expanding the content to include self-esteem, bullying, eating and nutrition issues, and HIV, along with practical self-help resources (eg, relaxation, social skills, stress management, and coping with negative thoughts). They also recommended using simple language to make the content more accessible for adolescents of different ages, reducing text, and adding more visual elements.

Following each session, the team systematically reviewed the feedback, identifying priority improvements and defining key technical or design challenges. Then, we developed an updated version of the chatbot that incorporated feasible recommendations while maintaining its core structure and objectives (Table 2). To enhance the chatbot’s presentation and navigation, an interactive web environment was created using the Wix (Wix.com Ltd) platform [40]. This transition gave the study team full control over the chatbot’s interface, enabling real-time error corrections during subsequent sessions with the YAB.

**Table 2.** Development of key features of the EVA (*Educación, Vinculación, y Autoayuda*) chatbot.

Feature	Version 0.1 (YAB^a^ Session 2)	Version 0.2 (YAB Session 3)	Version 0.3 (YAB Session 4)	Version 1.0 (YAB Session 5)
Chatbot presentation	No welcome screen: initially, the chatbot displayed an image of the EVA^b^ avatar along with a description clarifying that it was a chatbot, not a human.	Two welcome screens were created on the Wix platform: Screen 01 displays EVA’s avatar and a “Hello!” button, which can be clicked to proceed to the next screen, and Screen 02 introduces EVA through vignettes, images, and a video. Clicking the “Continue” button grants access to the chatbot.	Screen 01: improved by animating EVA’s full-body appearance and Screen 02: no change.	Screen 01: changed the “Hello!” button to “Start” and Screen 02: no change.
Modules built	Introduction: the chatbot greets users, asks for their name and age, and provides 3 options: Education, Linkage to Care, and Self-Help. Education: users can choose between 2 topics (depression and anxiety) or enter a custom query. The chatbot recognizes 8 keywords (anxiety, depression, sadness, treatment, HIV, healthy living, alcohol, stress) to provide more relevant information. Linkage to Care: if users opt to speak to a professional, a real-time chat is initiated. Self-Help: no content.	Introduction: the welcome greeting was simplified and made more conversational. Education: the module now introduces depression and anxiety, allowing navigation through buttons. Users are then presented with additional topics (stress and HIV) with submenus containing key information. Linkage to Care: users can now access this feature via a permanent button on the chatbot screen, which indicates whether a live agent is available. Self-help: no content.	Introduction: after entering information, users are redirected to a new “Introduction to Depression” module. Introduction to Depression: provides basic information about depression in a conversational manner. Once completed, users can navigate through the 3 modules via swipeable cards. Education: topics of depression, anxiety, and additional issues (stigma and treatment adherence) are now displayed on swipeable cards. Linkage to Care: the permanent on-screen button was removed. Instead, users can access options such as “Seek Professional Help” to start a real-time conversation or browse an emergency directory with contact information for crisis support. Self-Help: now includes 3 activities: deep breathing exercises, managing emotions (with 3 subactivities), and a game about facts and myths regarding HIV.	Introduction: no changes. Introduction to Depression: content was refined to be more concise, with fewer images and more accurate information. Emojis were added. Education: now includes eating disorders under additional issues Linkage to Care: the number of steps required to start a real-time conversation was reduced. Self-Help: a game about facts and myths regarding mental health in adolescents was added.

a YAB: Youth Advisory Board.

b EVA: *Educación, Vinculación, y Autoayuda*.

#### Session 3: Chatbot Interface Refinement and Assessment

For the third session, focus on reviewing the first improved version of the chatbot, highlighting updates made after initial feedback, including content integration, visual design adjustments, usability improvements, and correction of previously detected errors. At this stage, conduct a detailed and paced exploration of each selected chatbot section. Facilitators should guide navigation to ensure all components are reviewed and understood. Use simple guiding questions such as: “What do you think about this section? Is the information easy to understand? What would you change to improve it?” After reviewing each section, hold a brief group discussion before moving on to the next one. This structure allows feedback to remain specific and helps the group reach agreement on priority modifications related to navigation, presentation, and content.

**Figure 2.**
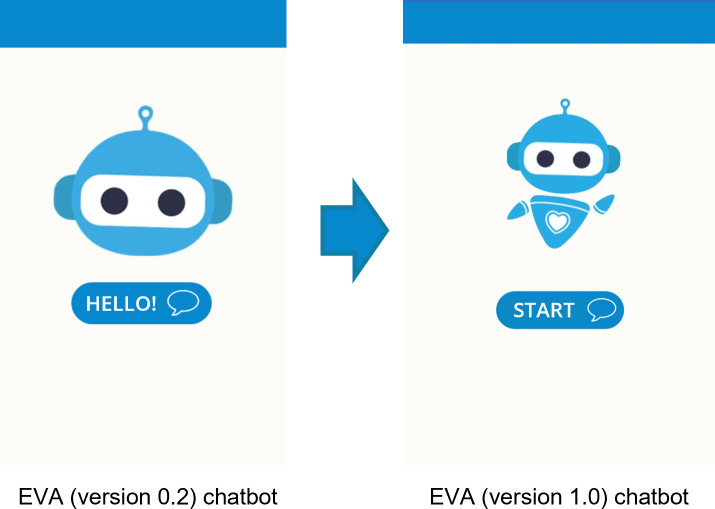
Improving first-screen usability through clear instructions, replacing the greeting (“Hello!”) with a direct-action cue (“Start”) to help users intuitively understand how to continue. EVA: *Educación, Vinculación, y Autoayuda*.

**Figure 3.**
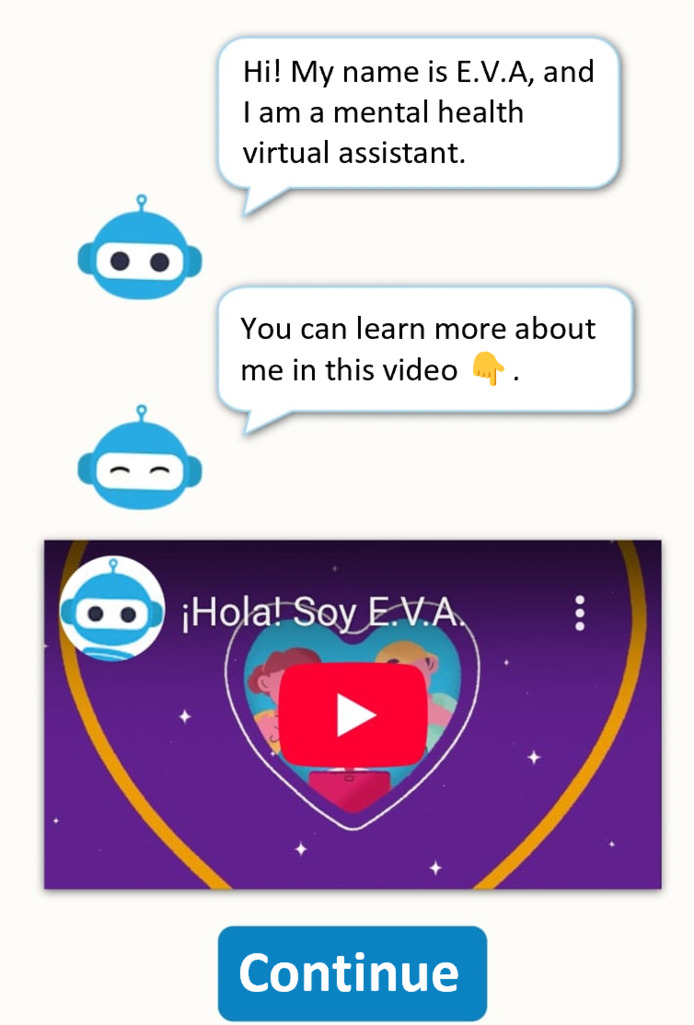
Audiovisual welcome screen to enhance early engagement. EVA (Educación, Vinculación, y Autoayuda) introduces itself as a virtual assistant and uses video and interactive elements to preview what users will experience.

In our project, each YAB member received blank sheets to record ideas and observations while reviewing each section of EVA (version 0.2). These notes were then used during the section-by-section discussions to help participants recall key impressions, compare perspectives, identify similarities and differences, and agree on priority changes for the next chatbot iteration. Participants found EVA (version 0.2) more visually appealing but noted that the initial screen was not intuitive, recommending clearer user instructions (Figure 2). The welcome message and introductory video—developed in the Wix web environment—were well received (Figure 3). While navigation buttons were considered useful, adolescents suggested limiting their number and using them mainly for simple choices, as excessive button use felt stressful, with a preference for more text-based interaction to increase engagement. They also reviewed 2 new videos created for the chatbot and recommended adding subtitles to all videos to improve accessibility and comprehension.

The “Education” module was viewed as clear and useful, though participants suggested a more engaging and optimistic tone, particularly for sensitive topics such as depression and anxiety. They further recommended including short, relatable stories about common adolescent experiences (eg, bullying, school stress, body image, eating concerns, HIV, and home situations) to make the content more meaningful.

Incorporating a quantitative method to monitor progress helps identify which chatbot features adolescents find most and least acceptable, enabling the team to track improvements and prioritize refinements. For EVA, a brief satisfaction scale using Likert-type items (1=very dissatisfied and 5=very satisfied) assessed the videos, the usefulness of information, content clarity, and engagement (Table 3). Additional items can be incorporated in later sessions to evaluate emerging features as the chatbot evolves. Results showed generally positive perceptions of usefulness and educational value, while lower ratings identified issues such as unintuitive design and repetitive navigation, both of which required further improvement.

**Table 3.** Summary of chatbot characteristics evaluated in Sessions 3 and 4.

Characteristic evaluated	Session 3, mean (SD; range)	Session 4, mean (SD; range)
Like on videos	4 (0; 4)	4.5 (0.55; 4-5)
Useful information	4 (0.45; 4-5)	4 (0.52; 4-5)
Clear information	4 (0.45; 4-5)	4 (0.41; 4-5)
Entertaining chatbot	3 (1.14; 1-4)	4 (0.75; 3-5)
Easy to use	3 (0.55; 3-4)	4 (0.52; 3-5)
Overall assessment	4 (0.45; 4-5)	4 (0.75; 4-5)
Self-help module utility	Not measured	4 (0.52; 4-5)
Use to seek help	Not measured	4 (0.52; 4-5)

#### Session 4: Chatbot Module Expansion and Usability Improvement

At this stage of development, once key design principles have been defined—such as appropriate text length, simple and age-appropriate language, the chatbot’s tone when addressing users, preferred input and interaction formats, and priority mental health topics—it becomes easier to expand the chatbot with new modules or content while maintaining coherence with its objectives. Teams should draft scripts for each new section to maintain a structured overview of content and flow, which facilitates revisions and consistency. When sharing scripts with adolescents, adapt them with visual aids (eg, illustrations or diagrams), especially when the text is extensive, as this supports comprehension and allows for more focused feedback. Reviewing scripts before implementation is particularly effective once participants are familiar with the chatbot’s structure.

For this step, prioritize accessibility, intuitive navigation, and engaging presentation. Introduce new ways of displaying information—even for previously reviewed topics—and invite feedback on these interaction formats. Since both the development team and YAB members become increasingly familiar with the chatbot over time, emphasize presenting new or updated content rather than repeating unchanged sections. Temporary navigation shortcuts (eg, quick-access buttons to specific modules) can help streamline testing sessions.

**Figure 4.**
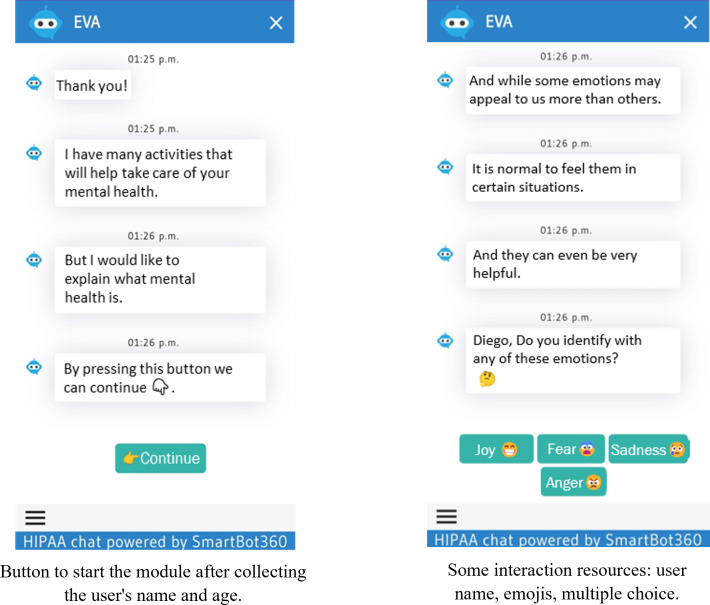
Introduction to Depression module. In a conversational way, it introduces and connects basic notions about emotions, mental health, and depression.

**Figure 5.**
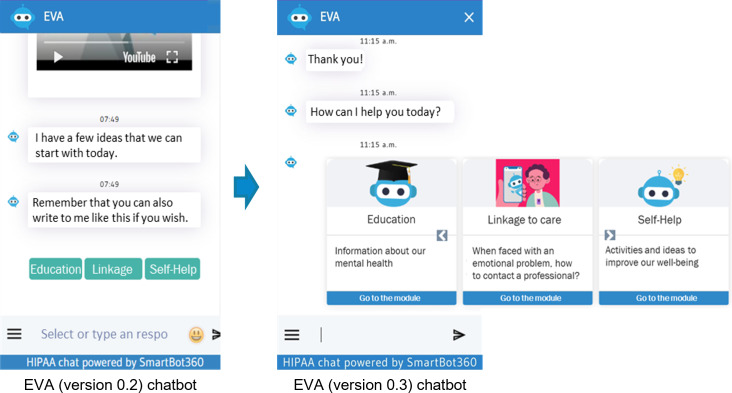
Changes in the presentation of EVA (*Educación, Vinculación, y Autoayuda)* chatbot modules. A cleaner and more pleasant appearance was achieved through shorter, more precise texts and improved visual resources (sliding cards).

During EVA (version 0.3) testing, quick-access buttons were added to the home screen to streamline navigation during the session. The YAB reviewed a new “Introduction to Depression” module (Figure 4), first annotating feedback on a printed script and then testing its functionality within the chatbot. Alternative navigation formats, including sliding cards, were introduced to improve content presentation and reduce repetitive button use (Figure 5), alongside updated self-help activities and new videos.

Participants reported that the interface felt more intuitive and preferred text-based interaction rather than repeatedly selecting buttons. The videos were well received, though members suggested incorporating additional visual elements. While the new module was considered useful, it was perceived as too lengthy. Extended use of the chatbot also revealed additional programming issues. The YAB recommended expanding educational topics (eg, eating disorders, bullying, and the roles of mental health care professionals). Satisfaction scores indicated improvements in navigation and engagement (Table 3). Final refinements focused on condensing content to enhance readability and resolving remaining technical issues.

#### Session 5: Final Review and Validation

In the final validation session, present the chatbot in a format that closely reflects how a new user will experience it. Remove temporary testing features such as quick-access buttons or shortcuts and allow adolescents to navigate the tool under realistic conditions. Provide sufficient time for free exploration of all content; if the material is extensive, organize the review by modules or sections while maintaining a natural flow of use.

After each review segment, collect adolescents’ feedback. Because participants are already familiar with the chatbot, facilitators should probe more deeply into their responses, asking why they hold certain opinions or suggest specific changes. Comparing the current version with earlier prototypes can help identify which modifications were most positively received and which areas may still need refinement.

Validation is considered achieved when YAB members show broad agreement that the content, tone, and functionality are appropriate for the chatbot’s intended purpose and future implementation. To support this, clearly explain how the chatbot will be used in the next phase of the project. If significant suggestions or unresolved issues remain, schedule an additional review session before finalizing the tool.

**Figure 6.**
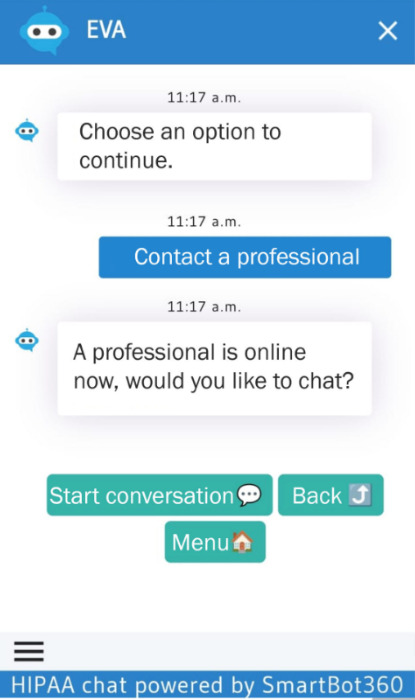
Linkage to Care module screen. EVA (*Educación, Vinculación, y Autoayuda*) shows whether a professional is available and provides buttons to start a chat or return to previous or main menus.

The YAB approved the overall interface and audiovisual materials, suggesting only minor navigation improvements, such as adding text labels (eg, “Back”) to icon-only buttons to make navigation more intuitive. They responded positively to the shortened “Introduction to Depression” module, noting that it delivered key information without overlapping with other content in the “Education” module. The self-help activities were found to be engaging and clear, particularly an interactive emotion-regulation story. The “Linkage to Care” module was also considered easy to access and use (Figure 6).

Including multimedia components is strongly recommended when developing digital tools for adolescents, as brief audiovisual materials can enhance engagement, comprehension, and information retention, particularly when addressing sensitive topics. Evidence from digital health interventions [21,41] shows that short, structured multimedia content improves user engagement and perceived usefulness in youth-focused eHealth tools. Accordingly, videos integrated into chatbots, or similar applications, should be concise (ideally under 1 minute), use clear and age-appropriate language, and present relatable scenarios or examples.

From a user-centered design perspective, decisions about audiovisual tone, style, and priority topics should be explored in early stages and validated with adolescent advisors before chatbot development begins. Because chatbot content often evolves during development, a practical first step is to create a short introductory video that explains the chatbot’s purpose, structure, and main modules. The design, characters, and visual style defined in this first video are crucial, as they establish the aesthetic and narrative coherence for subsequent audiovisual materials. As content is defined, additional short videos can be developed to introduce key concepts, explain unfamiliar terms, or highlight essential features, such as self-help strategies or linkage to professional care, ensuring alignment between multimedia elements and the tool’s overall objectives.

For EVA, an audiovisual consultant produced 5 animated videos of approximately 30 seconds each (Table 4), based on scripts and visual guidelines from the research team, which were subsequently reviewed with the YAB. The animation style was selected following adolescent consultation and in consideration of the broad target age range (10‐19 years) to ensure relevance and acceptability across developmental stages. The videos were stored in a dedicated YouTube (Google LLC) [42] account and embedded within the chatbot, allowing users to view them directly through the chatbot interface.

**Table 4.** Videos developed for the EVA (*Educación, Vinculación, y Autoayuda*) chatbot.

No	Video title (Spanish)	Chatbot location	Main objective
1	Hello! I am EVA (*“¡Hola!, soy EVA”*)	Welcome screen	Introduce the chatbot and its structure.
2	Self-Help (*“Autoayuda”*)	Self-Help module	Explain the purpose of the self-help section.
3	Why seek help? (*“¿Por qué buscar ayuda?”*)	Linkage to Care module	Promote help-seeking behavior.
4	Adherence (*“Adherencia”*)	Education module	Highlight the link between mood and health behaviors.
5	Stigma (*“Estigma”*)	Education module	Address stigma related to HIV and mental health.

### Step 3: Testing Before Launch

An external review by professionals who were not involved in the development process is recommended to mitigate familiarity bias that may emerge as the development team and adolescent advisors become accustomed to the tool during iterative construction. Methodological research on co-design development highlights the importance of involving diverse stakeholders to enhance content validity, detect blind spots, and strengthen credibility before implementation [43-45]. In digital health design, incorporating external expert review is also considered a strategy to improve rigor, transferability, and content accuracy. Ideally, these reviewers should have experience with the target population and the intervention’s thematic focus, as this allows feedback not only on usability but also on clinical relevance and appropriateness.

In our study, after YAB approval of EVA (version 1.0), 20‐30-minute test sessions were conducted with 6 external HIV health care professionals (4 physicians, 1 nurse, and 1 psychologist) working with adolescents living with HIV. Their feedback led to simplifying navigation elements (eg, clearer button labels, shorter welcome messages, standardized “Continue” buttons), refining slide-out menus, and reducing textual redundancy. The “Introduction to Depression” module was reframed to begin with emotions rather than abstract mental health definitions to enhance relatability. Content was further tailored to adolescents living with HIV by condensing general prevention information and expanding topics such as sexual health, relationships, HIV transmission, and treatment adherence. Final internal testing sessions were then conducted to verify functionality and content coherence before proceeding to formal feasibility and acceptability evaluation with a new sample of adolescents.

## EVA’s Final Version: System and Modules Structure

**Figure 7.**
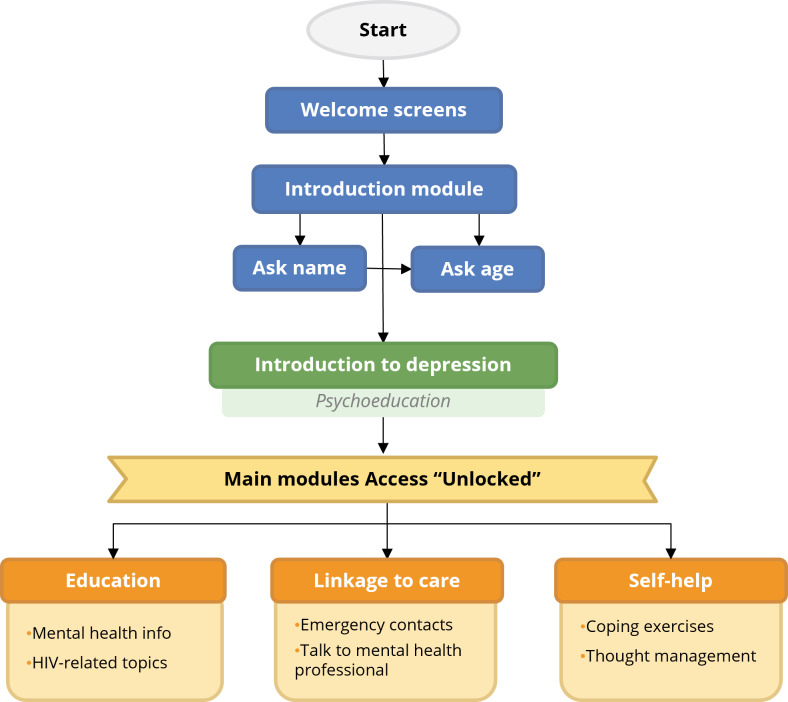
Structure and user flow of the EVA (*Educación, Vinculación, y Autoayuda*) chatbot.

A final chatbot should integrate the core modules identified through user research and iterative testing, organized into a clear navigational structure. For EVA, the chatbot was developed using Smartbot360 and structured into 5 sections: “Introduction,” “Introduction to Depression,” “Education,” “Linkage to Care,” and “Self-Help” (Figure 7). A final video that describes and promotes EVA was also produced (refer to Multimedia Appendix 1).

After 2 welcome screens featuring an introductory video, the chatbot prompts the user for their name and age. Age is collected to confirm eligibility within the target range of 10‐19 years, detect out-of-range users, inform health care professionals in an eventual follow-up, and for future analyses. The “Introduction to Depression” module provides brief, adolescent-friendly information on mental health and depression. It is displayed only during the user’s first access to the chatbot, after which full access to the core modules is granted.

The “Education” module covers depression, anxiety, eating disorders, HIV-related topics, treatment adherence, and discrimination. The “Linkage to Care” module includes emergency contacts and a text-based feature to connect with health care professionals. The “Self-Help” module offers interactive activities focused on relaxation, emotional regulation, and myth clarification related to HIV and mental health.

When budgeting for chatbot development, teams should account for platform subscription fees, multimedia production, personnel time, and logistical costs for participatory sessions. For EVA, development costs included Smartbot360 subscription (US $99/month for up to 10,000 messages) and production of 5 animated videos (US $2710). Personnel time and logistical expenses related to YAB sessions (eg, refreshments, transportation, and incentives) were excluded from this estimate. Teams should develop a detailed budget early in the planning process, as actual costs may vary significantly depending on design complexity, content volume, and the inclusion of additional human resources.

## Lessons Learned

This tutorial provides a step-by-step guide to the recommended and implemented decisions during the development of the EVA chatbot.

First, actively involve adolescents from the earliest conceptual stages. Early qualitative interviews, focus groups, and the creation of a YAB ensured that the chatbot’s tone, structure, aesthetics, and priority topics reflected adolescents’ lived experiences. Similar participatory approaches have been used in the development of mental health chatbots such as [32], Woebot for Teens (Woebot Health) [21], and Zuri [46], underscoring the role of co-design in cultural relevance, acceptability, and engagement. Prior qualitative exploration is particularly useful to define conversational style and interface decisions before programming begins [47-49]. Transparent discussions about technical constraints, such as limitations in advanced artificial intelligence (AI), are also recommended to maintain trust and realistic expectations [21,35,50].

Second, adopt an explicitly iterative development model. Continuous feedback cycles led to improvements in readability, emotional tone, and module length in response to adolescents’ preferences for autonomy and brevity [47]. Such user-driven refinement is essential, as adolescents must perceive the intervention as not only useful but also motivating and hopeful, particularly when discussing sensitive topics such as depression and HIV-related stigma. These principles align with iterative testing approaches described in chatbots such as Wysa (WYSA Ltd) and Woebot (Woebot Health) [21,45,46]. Ensuring an encouraging, supportive, and culturally sensitive tone aligns with evidence that highlights the importance of fostering optimism and self-efficacy in adolescent digital interventions [21,51].

Third, tailor content to the specific psychosocial context of the target population. EVA’s “Introduction to Depression” module, for instance, uses destigmatizing language to normalize conversations about emotional distress and reduce barriers to help-seeking, reflecting the reality that many adolescents living with HIV face limited family support and restricted access to reliable mental health information due to socioeconomic constraints. Comparable design choices have been described in chatbots such as Zuri in Kenya [46]. Framing depression through emotions and common symptoms, rather than solely as a clinical condition, promotes a more accessible understanding of mental health and may strengthen adolescents’ motivation to seek care.

Finally, maintain direct control over the development tools whenever feasible. The ability to implement real-time corrections facilitates immediate responses to YAB feedback and transparent dialogue about technical possibilities and limitations. Similar hands-on, low-cost development models have been reported in LMICs, where research teams favored flexibility and transparency over sophisticated automation [21]. Involving health care professionals with expertise in HIV and adolescent mental health validated and refined EVA’s content, ensuring accuracy, clinical relevance, and practical utility [48].

## Costs and Sustainability

The EVA development used relatively low-cost resources: an affordable messaging platform, free web hosting, and open-access images, demonstrating the feasibility of building a functional mental health chatbot in LMICs. Overall costs will vary significantly depending on the complexity of the design, the software required, the audiovisual production quality, and the volume of content included. Comparatively, digital mental health chatbots that integrate advanced AI require substantial investments—particularly when integrating regulatory compliance or large-scale technical deployment—and may even reach tens of thousands of dollars for full development [21,41,52]. In contrast, development teams can minimize costs by foregoing specialized technical staff and relying on free hosting and basic content production.

Nevertheless, replications or expanded versions of this study may require considerably higher budgets if professional developers, graphic designers, robust web infrastructure, or professional audiovisual production are included. Additionally, other costs—such as hiring part-time support staff, organizing meetings with new YAB members, providing incentives, transportation, and testing devices—would only be relevant if the participatory phases of the study are explicitly replicated. Thus, the planning of similar initiatives in LMICs should account for a baseline of moderate costs based on functional platforms and accessible content, while also preparing scalable scenarios for more complex versions that integrate professional technical development, secure infrastructure, and expanded community participation.

## Challenges and Limitations

Practitioners should anticipate several challenges when developing similar tools. Although participatory approaches and iterative testing with a YAB can yield significant improvements, some limitations—such as software constraints and an initial reliance on button-based navigation—may not fully meet adolescents’ desire for more advanced, AI-driven conversations found in freely available digital products they are already familiar with. Future efforts should explore more sophisticated platforms that offer greater flexibility, interactivity, and personalization, as well as improved data collection methodologies to track and prioritize user feedback systematically. Standardizing evaluation methods across development stages and expanding content on related issues, such as bullying, eating disorders, and distinctions between mental health care professionals, may increase a chatbot’s relevance and uptake.

## Conclusions

This tutorial describes the step-by-step development of a mental health chatbot through sustained collaboration between adolescents, researchers, and clinicians. The process illustrates how structured adolescent engagement, clear goal-setting, and iterative refinement are fundamental to producing digital tools that are acceptable and feasible for end users. Through continuous feedback cycles and validation by mental health and HIV specialists, EVA achieved a balance between scientific accuracy, emotional resonance, and cultural sensitivity.

Our experience also highlights that participatory, low-cost, and contextually grounded design processes can produce relevant and sustainable digital interventions in resource-limited settings. Future versions of similar tools may incorporate more advanced conversational features, digital assent mechanisms, and scalable infrastructure to enhance sustainability. Although challenges remain, the lessons presented throughout this tutorial provide valuable guidance for researchers and practitioners seeking to develop adolescent-centered digital health interventions, contributing to improved quality, accessibility, and impact of digital support for adolescents living with HIV and beyond. We encourage teams to adapt the steps outlined here to their own contexts and populations, using participatory methods to ensure that the tools they create are both evidence-informed and responsive to the needs of the communities they serve.

## Supplementary material

10.2196/75351 Multimedia Appendix 1 Video made at the end of the study to explain the content and operation of the EVA (*Educación, Vinculación, y Autoayuda*) chatbot. Reproduced from Socios En Salud [53].

## Abbreviations

AI: artificial intelligence
ART: antiretroviral therapy
EVA: *Educación, Vinculación, y Autoayuda*
HCD: human-centered design
IRB: Institutional Review Board
LMIC: low- and middle-income country
PHQ-9: Patient Health Questionnaire-9
UNAIDS: Joint United Nations Program on HIV/AIDS
WHO: World Health Organization
YAB: Youth Advisory Board
